# Food Security beyond Cereals: A Cross-Geographical Comparative Study on Acorn Bread Heritage in the Mediterranean and the Middle East

**DOI:** 10.3390/foods11233898

**Published:** 2022-12-02

**Authors:** Dauro Mattia Zocchi, Camilla Bondioli, Seyed Hamzeh Hosseini, Mohamed Djamel Miara, Carmelo Maria Musarella, Datis Mohammadi, Ajmal Khan Manduzai, Kovan Dilawer Issa, Naji Sulaiman, Chadi Khatib, Hiwa M. Ahmed, Tola Abdulsattar Faraj, Hawraz Ibrahim M. Amin, Faiq H. S. Hussain, Abdullah Faiz, Antonella Pasqualone, Frits Heinrich, Michele Filippo Fontefrancesco, Andrea Pieroni

**Affiliations:** 1 University of Gastronomic Sciences, Piazza Vittorio Emanuele 9, 12042 Pollenzo, Italy; 2 Department of Biology, Faculty of Science, University of Jiroft, Jiroft P.O. Box 78671-55311, Iran; 3 Agrobiotechnology and Nutrition Laboratory in Semi-Arid and Arid Zones, Department of Nature and Life Sciences, Ibn-Khaldoun University, BP P 78 Zaâroura, Tiaret 14000, Algeria; 4 Department of Agriculture, Mediterranean University of Reggio Calabria, Via dell’Università, 25 (Già Salita Melissari), 89124 Reggio Calabria, Italy; 5 Department of Environmental Sciences, COMSATS University, Abbottabad Campus, University Road, Abbottabad 22060, Pakistan; 6 Department of Medical Analysis, Faculty of Applied Science, Tishk International University, Erbil 44001, Iraq; 7 Department of Crop Sciences and Agroforestry, Faculty of Tropical AgriSciences, Czech University of Life Sciences Prague, Kamýcká 129, 165 00 Prague-Suchdol, Czech Republic; 8 Department of Pharmacognosy, Faculty of Pharmacy, Damascus University, Damascus P.O. Box 30621, Syria; 9 Sulaimani Polytechnic University, Slemani 46001, Kurdistan Region, Iraq; 10 Department of Horticulture, College of Agricultural Engineering Science, University of Raparin, Ranya 46012, Kurdistan Region, Iraq; 11 Department of Basic Sciences, College of Medicine, Hawler Medical University, Erbil 44001, Iraq; 12 Department of Chemistry, College of Science, Salahaddin University-Erbil, Erbil 44001, Iraq; 13 Department of Medical Biochemical Analysis, Cihan University-Erbil, Erbil 44001, Iraq; 14 Faculty of Agriculture, University of Herat, Herat 3001, Afghanistan; 15 Department of Soil, Plant and Food Science, University of Bari Aldo Moro, Via Amendola, 165/A, 70126 Bari, Italy; 16 Research Group Social and Cultural Food Studies (FOST), Department of History, Vrije Universiteit Brussel, Pleinlaan 2, 1050 Brussels, Belgium; 17 Research Group Industrial Microbiology and Food Biotechnology (IMDO), Department of Bioengineering Sciences, Vrije Universiteit Brussel, Pleinlaan 2, 1050 Brussels, Belgium; 18 Department of Anthropology, Durham University, Stockton Road, Durham DH1 3LE, UK

**Keywords:** balanophagy, food security, food heritage, food scouting, ethnobotany

## Abstract

This article aims to contribute to the limited literature on traditional gastronomic knowledge concerning acorn-based bread by ethnographically documenting the ingredients, preparation techniques and consumption practices of baked goods made from acorn seeds and flour that are still used today or at least still present in living memory. A qualitative comparative case method was adopted, and ethnographic data were gathered from 67 people in six selected Mediterranean, Central Asian and Middle Eastern countries. The analysis highlighted distinct trajectories in the development of acorn-based bread, showing some differences in terms of ingredients, preparation techniques and baking methods in the two cultural and geographical macro-regions. By exploring the evolution of the alimentary role of acorn bread in the past century, our findings also support the hypothesis that the product, at least during the last two centuries, has mostly been used as a famine food. By acknowledging the cultural importance of acorn fruits and acorn-based products, this study suggests that the rediscovery of acorn-based products and associated traditional knowledge may foster the sustainable development of rural and marginal regions in the Mediterranean, Middle East and Central Asia. This could help to reinforce the resilience of local communities and thus increase food security. Furthermore, reassessing acorns as a foodstuff may aid in developing innovative products in line with emerging trends in the food sector, which is looking for new non-cereal-based bakery products and other novel culinary applications.

## 1. Introduction 

*Quercus* spp. (family Fagaceae) is an important genus consisting of both evergreen and deciduous trees that occur in both temperate and tropical climatic zones. From early prehistory onwards, the fruits of different species belonging to this genus were part of the traditional livelihoods and foodscapes of communities worldwide [1].

While often perceived as animal feed, with herds of livestock pigs being released into oak forests to browse for acorns (a widespread practice referred to as pannage [2], acorns represented an important part of both the gastronomical and medical folklore of various regions of North America, Europe, North Africa, the Near East and Central Asia, as indicated by a wealth of ethnoarchaeobotanical and historical evidence [3,4,5,6,7,8,9,10,11,12,13,14,15,16,17,18,19,20,21,22,23,24,25,26].

Rural populations used to collect and eat acorns from different *Quercus* species. While sweet acorns (e.g., *Quercus pubescens* Willd. subsp. *pubescens*) were eaten directly [21], astringent and bitter acorns (i.e., fruits with a high tannin content, such as *Quercus rotundifolia* Lam.) were processed through heating, leaching [27,28,29] or complex detoxification techniques involving the use of clay [30,31,32]. In addition to the consumption of roasted and boiled acorns, either in porridge or as an ingredient in a variety of dishes, seeds were often ground into flour and used in the preparation of different types of bread, especially in the Mediterranean, Central Asia and the Middle East [8,19,33,34,35].

Until the first half of the twentieth century, the human consumption of acorns, also known as balanophagy (see [4]), played an important role in the food security of rural Mediterranean, Central Asian and Middle Eastern populations, providing an affordable and nutritionally rich source of carbohydrates, proteins and fat [29,36]. Today, this practice has declined, and the traditional knowledge related to the use and processing of acorns has been eroded and, on several occasions, apparently lost [19,37]. In this regard, archaeologists, historians, ethnographers and ethnobotanists have explored the dietary role of acorns and the past use of acorn seeds for the preparation of bread, highlighting some of the reasons behind the marginalisation of acorn and acorn-based products in the present diet. However, only sporadic attention has been paid thus far to the ethnographic documentation of the traditional knowledge and practices associated with the production and consumption of acorn-based foodstuffs, as well as to the modern continuation of these practices and the associated aspects of food and cultural heritage.

On the basis of these premises, this study records the uses and importance of acorn-based bread and baked goods by comparing the traditional practices and associated knowledge linked to the preparation and consumption of these products in selected Mediterranean, Middle Eastern and Central Asian countries. Therefore, the aims of this study are (1) to ethnographically document ingredients, preparation techniques and consumption practices pertaining to baked goods made from acorn seeds and acorn flour that are still used today or at least still present in living memory; (2) to explore the possible evolution of the alimentary role of these products and associated practices in the past century, identifying the main drivers behind any observed changes; (3) to analyse, from a geographically comparative perspective, similarities and differences regarding the preparation and consumption of these products, as well as the dynamics underpinning the conservation or erosion of the traditional gastronomic knowledge associated with these practices.

By acknowledging the importance of acorn fruits in the cultural heritage of local communities and their potentially crucial application as an alternative solution for the future of rural economies, this study aims to open a debate on the potential effects that the revitalisation of acorn-based products and associated foodscapes may have on the sustainable development of rural and marginal regions in the Mediterranean, Central Asia and the Middle East.

## 2. Background

### 2.1. Archaeological and Historical Evidence for the Consumption of Acorn Bread

While the collection and use of acorns are widely archaeobotanically attested, it is often difficult to confirm their use for human consumption, let alone their use for specific food items. However, microbotanical acorn remains on rotary querns used for grinding flour, or the presence of acorn-derived compounds in residues found in vessels or other cooking contexts may be helpful in this respect [18,26,38,39,40].

The culinary use of acorns in bread and other preparations has also been documented in ancient historical sources, whose authors were well aware of the differences between sweeter and more bitter types. Theophrastus (371–287 BC), in his *Historia Plantarum*, recounts that the fruits of the Valonia oak are the sweetest and that oaks growing in the mountains and wild lands typically have sweeter fruits than those near cultivated land (see [41], cf. [42]). An important bias in many ancient descriptions is that these texts were invariably written for and by members of the elite, while the consumption of acorns was a low-status activity and, therefore, was very much underrepresented. Misrepresentation is a potential issue as well. While oak trees themselves were held in high esteem, often serving as sacred or royal trees [43], the eating of acorns was often associated with being rustic and less civilised. This could have a positive connotation, with the consumption of acorns being representative of the mythic Golden Age of hunting and gathering, before the toil of agriculture [8,44], as it is apparent, for instance, in the description of early humans by Lucretius (5.939) and Ovid (*Fasti* 4.395-402). Similarly, in reference to the inhabitants of Arcadia (a region of Greece having the reputation of being rustic throughout antiquity), the Arcadians are described as ‘stout acorn eaters’ (Herodotus 1.66—cf. Pausanias 8.1.6 and Galen *De alimentorum facultatibus* II. 38). Acorn consumption could also be represented as being more barbaric or antiquated, as ancient ethnographers often highlighted or exaggerated perceived differences with their own culture. Examples of this can, for instance, be found in the *Anabasis*, a book by the Greek mercenary general Xenophon (ca. 430–355 BC) about the Mossynoecians, people living in a region on the southern shores of the Black Sea in present-day Turkey. He writes that they stored large quantities of acorns on the upper floors of their houses (likely for drying) that were boiled and then baked afterwards into a type of bread that was the main part of their diet. Another example can be found in the *Geographica* of the Greek geographer Strabo (c. 64 BC–AD 24), in which he describes the Lusitanians on the Iberian peninsula as those living in the mountains subsisting on acorn seeds for two-thirds of the year and—after the acorns are dried and ground—are made into bread that ‘may be stored away for a long time’ (Strabo 3.3.7). Pliny the Elder (c. AD 23–79), in his *Naturalis Historia*, reflects on the importance of acorns as a food source for many peoples ‘even when enjoying peace,’ adding that especially in times of cereal shortages and famine, acorn seeds are dried and ground into a flour from which bread is made. Pliny also mentions that roasting acorns in ash makes them sweeter. It is suggested that acorns were important in the early history of Rome as well, as Pliny refers to ancient Roman laws discussing the permissibility of collecting acorns from land owned by others (*NH* 16.6–9), although some have argued that by Pliny’s time, this would have only applied to famines [45].

Famine and shortage are recurring themes when acorns are mentioned as a food source for humans in classical sources; for instance, the poet Hesiod (ca. 750–650 BC) wrote that Zeus staved off famine for the just, and for them ‘on the mountains the oak tree bears acorns on its surface’ (*Works and Days* 225–237). Often such instances represent a commodity that would normally serve as animal fodder being turned into human food [46]. In a book on the properties of different foodstuffs, the Roman physician Galen (AD 129–199) from the city of Pergamon in what is now Turkey described how local farmers would normally collect acorns as pig feed (mast) for winter, but when a bad harvest and famine struck the area was suspected, the farmers slaughtered and ate their pigs and stored the acorns in pits and storage vessels that were otherwise used for cereals. The acorns would be boiled and sometimes covered in hot ashes to bake them, while in other cases, they were crushed and made into porridge or soup to which milk and even honey could be added (*De alimentorum facultatibus* II. 38, cf. [47,48]). Although slow to digest (cf. Hippocrates *Regimen* 55), Galen notes that acorns were just as nutritious as cereals and much more nutritious than other wild plants (*De alimentorum facultatibus* II. 38, cf. Artemidorus Daldianus, *Oneirocritica* 2.25, [49]). The image of acorns as a famine food that would only delight mythic ancestors, backwards rustics and quaint foreigners is not entirely fair. There are, for instance, also references to the use of acorns in *de Re Coquinaria*, a corpus of recipes that was likely compiled in the fifth century AD and fictitiously attributed to the first century AD Roman gourmet, Apician. Acorn seeds are, for example, used in a recipe for stuffed hare (*Apicius* 8.8.3). Ancient attitudes towards the status of acorns as a foodstuff may show similarities to what has been observed for pulses and ‘inferior’ cereals; it is not that these foodstuffs are seen as intrinsically bad or low status themselves, but rather it is the reliance on them as staples that is viewed negatively [50,51].

In the (Mediaeval) Islamic world, acorns were known as food as well, although literary sources are somewhat scarce. The Persian philosopher and physician al-Razi (ca. AD 845-925/935), for instance, mentions acorns as a potential ingredient for cheap *mulla* bread in his work *Manāfiʻ al-aghdhiya wa-dafʻ maḍarrihā* (for a discussion, see [52]). When describing the region of Bilad as-Sham (Greater Syria), the Palestinian geographer Al-Muqaddasi (ca. AD 945–1000) mentions that people in the Jabal al-Jawlan region of Syria consumed acorns which they split, let dry to sweeten and then ground and mixed with ‘wild’ barley—although it is not mentioned if this flour was used for bread or porridge (Al-Muqaddasi, p. 188, translation [53])). The Arab-Nestorian Christian physician Ibn Buṭlān (AD 1001–1064), in his medical work *Taqwīm aṣ-Ṣiḥḥa*, mentions that fresh acorns were healthy, although women should eat them roasted and with sugar to prevent adverse effects to menstruation (see [54]). In thirteenth-century Islamic Iberia (al-Andalus), a more elite and culinary reference to acorn use is found. In the cook book by Ibin Razīn al-Tujībī (AD 1227–1293), there is a recipe for fried chicken pieces coated with egg (III. 2.43), in which fresh acorns (without drying) are used alongside chestnuts and boiled, after having been shelled and blanched (see translation [55]). In Mediaeval European sources, acorns were increasingly referenced as pig feed. However, they were also mentioned as penance food, an item used by those living off the land, and again as famine food. Drying, shelling and subsequently grinding acorns into a flour that could be mixed with flours made from millet, buckwheat or chestnuts is widely attested, especially in Italy, where such flours appear to have been used to make *polente* and *necci* (flatbread) [54]. As Maraschi [54] observes, from the sixteenth century onwards, there appears to be a brief episode in culinary history during which acorns became fashionable in the cooking of the Italian elite, with boiled acorns being used in pies, among other dishes.

### 2.2. Ethnographic Evidence for the Consumption of Acorn Bread

Throughout the Mediterranean basin, acorn flour and bread have been used for centuries by rural and pastoral communities as a staple food, especially during times of famine [8,37]. In the aftermath of the Spanish Civil War, acorn bread was an important food in the diet of the inhabitants of the Iberian Peninsula, especially in the southern part of the Spanish *dehesa* [2], Cantabria [56], Extremadura, Castilla-La Mancha [57] and the Basque Country [19,58]. Such uses of acorns were also documented for Greece, particularly for the Argolid and wider Peloponnese [3,59].

The consumption of acorns in the form of bread has been reported in ethnobotanical and ethnographic studies conducted in the central and southern regions of Italy [60,61,62,63]. Until the first half of the twentieth century, the practice of mixing acorn flour with cereal flour was common in rural areas of Umbria and Tuscany [7], Marche [64], and Basilicata and Calabria [28]. Moreover, the use of acorns in the preparation of bread has been recorded in Sardinia, especially in the Ogliastra subregion of Nuoro province [30,65,66,67,68], where a particular type of bread (*pan’ispeli*) made from water, acorn seeds, and ash and clay (rich in ferrous elements), was consumed until around the beginning of the twentieth century [65]. The use of clay and ash to remove tannic acid from acorns is also documented in North America, especially in the western region of the continent, where sweetened bitter acorns were an important staple food among Native American communities [7,30,69,70]. The Pomo Indians of California made dark bread by preparing dough with ground acorns, red clay and water and then baking it in underground ovens [30].

In the Mediterranean African countries, especially Algeria and Morocco, flour made from acorns was used in times of food shortage as a substitute for wheat and other grains in the production of bread, cake and pastries [34,71,72]. In the Algerian region of Chenoua, acorn flour, in particular, was used for the preparation of *taâm oubeloout* (acorn-based couscous), especially during the French colonial period [73].

In the Middle East, as well, rural pastoral communities in mountainous areas have utilised acorn seeds for different alimentary purposes, including the production of flour and baked goods [27]. These practices have been documented in Turkey [33], Iraq [74,75], Israel [76], Jordan [77] and Iran [35]. Drawing on the analysis of different literary sources, Potts [35] provided evidence for the use of acorn flour for the preparation of flatbreads and cakes in southern and western Iran (especially in the provinces of Kurdistan and Luristan), particularly when other resources were lacking. Due to poverty and the inadequate supply of cereals, pastoralists used to satisfy the alimentary requirements of their households by gathering acorns and making bread with pure acorn flour (*kazqa*) or by mixing it with wheat flour (*kalg*). Potts [35] argues that the modern usage of acorns (i.e., in the twentieth century) reinforces the hypothesis that acorns were a staple food prior to agriculture.

## 3. Materials and Methods

The research conducted in the present study is based on a qualitative comparative case method [78] carried out through the food scouting approach, i.e., trans-disciplinary ethnographic-based research methods aimed at mapping, inventorying and documenting food and gastronomic elements embedded in local and traditional foodscapes [79,80]. Specifically, several eco-gastronomic units of concern (i.e., local food biocultural heritage resources threatened or endangered [81]) were explored to document the traditional practices and knowledge linked to the collection of acorns (*Quercus* spp.), the processing of seeds into flour and the preparation of baked goods, as well as to understand the driver(s) underpinning their evolution. For these purposes, we have collected data on the use of acorns for breadmaking through ethnographic research conducted by different research teams. For each unit, individual access was negotiated with the analysis based on the specific configuration of the locally available oral and written sources.

### 3.1. Study Areas

**Figure 1:**
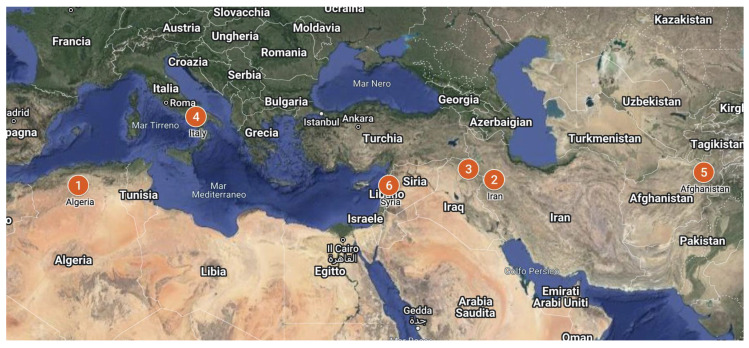
Map showing the study area (File credits: Creative Commons Attribution-Share Alike 3.0 licence). Key: 1. Algeria; 2. Iran, 3. Iraq; 4. Italy; 5. Afghanistan; 6. Syria.

Fieldwork was conducted between 2020 and 2022 in select Mediterranean, Central Asian and Middle Eastern countries. Research activities were carried out in the following countries to account for variations in local traditions and practices of acorn-based breadmaking: Algeria, Iran, Iraq, Italy, Afghanistan and Syria (Figure 1).

The key locations of the study were specifically chosen to focus on regions where there was past evidence of the preparation of acorn-based bread and baked goods. To this end, we relied on an analysis of scientific and grey literature sources, as well as personal communications and observations collected by members of the research team involved in this study.

Despite the geographical, cultural and socioeconomic differences between these countries, they have previously been reported as areas where acorns have been used, mostly in times of famine and scarcity, for alimentary purposes and, specifically, for the preparation of bread and baked goods [27,28,35,68]. In these areas, where pastoral and agro-silvopastoral systems have traditionally been the main source of livelihood for rural populations, acorn trees (*Quercus* spp.) have played a vital role as a source of food, medicine, animal fodder and timber.

### 3.2. Fieldwork, Data Collection and Analysis

Ethnographic data were gathered through personal observations, informal conversations and semi-structured interviews with 67 people (33 men and 34 women). Informants were selected via convenience sampling with the constraint that they were elderly community members who, during their lives, were more likely to have prepared, consumed or at least remembered acorn bread and the associated preparation techniques. Table 1 lists the exact research locations and the sociodemographic characteristics of the individuals involved in this study.

By drawing on the personal knowledge and experience of the informants, the fieldwork was aimed at collecting information on the following topics: acorn species used in the preparation of bread and baked goods, the utilised processing methods (e.g., the debittering process of seeds and the production of flour), bread making techniques and methods, as well as the traditional consumption practices associated with these products. Particular attention was paid to the perceived changes in the alimentary role and socioeconomic value of acorn-based products and the main reasons underlying these phenomena.

To this end, C.B. carried out interviews among the inhabitants of the Ogliastra subregion (Nuoro province) in Sardinia, Italy (*n* = 8); C.M.M. gathered data in three different provinces of Calabria, Italy (*n* = 6), while N.S. and C.K. gathered data in Syria (*n* = 2) and M.D.M. in Algeria (*n* = 5). F.H.S.H., T.F., K.D.I. and H.M. carried out fieldwork in Iraqi Kurdistan (*n* = 5), while H.I.H.A. and D.M. conducted fieldwork in the Kurdistan region and eastern part of Iran (*n* = 31). A.F. and co-workers gathered data from the eastern part of Afghanistan (*n* = 10). Finally, A.K.M. conducted an exploratory survey among the Afghan diaspora in the Mansehra, Khyber Pakhtunkhwa province, Pakistan (*n* = 41).

Interviews were conducted by the authors (see paragraph above) in Italian (Sardinia and Calabria), Arabic (Syria and Algeria), Kurdish (Iraq), Lori and Kurdish (Iran) and Dari (Afghanistan). Before each interview, informed consent was obtained from each informant, as recommended by the International Society of Ethnobiology code of ethics [82]. The project's rationale, aims and expected outcomes were explained in advance.

The interviews and the field notes were translated into English, transcribed and entered into NVivo version 12.5.0 [83], and then codes, concepts and nodes were generated during the qualitative data analysis. All the data were organised and subsequently selected and condensed as tables and compared in order to (1) highlight the different ingredients, steps and operations linked to the production of acorn-based bread and baked goods; (2) understand the past and present role of these products in the local food culture and their associated gastronomic value; and (3) identify the reasons behind the abandonment or continued use of these products.

Species identifications were made by the authors (ethnobotanists with extensive knowledge of the local floras) in the field. For those plants for which specimens were not available, probable identification was obtained by asking the interviewees to describe the plant and its habitat. For botanical nomenclature, we followed the criteria set by Plants of the World Online [84]. All local plant names were transcribed from the recorded local languages using the Latin alphabet.

## 4. Results

For each of the surveyed areas, field data were organised into tables that outline the following information: product category, local/vernacular names, acorn species used in the preparation, ingredients, harvesting and post-harvesting phases and processing methods/techniques. A summary of the main findings of our study is reported in Table 2.

In the following sections, the most relevant results of the study are outlined with a focus on the traditional practices linked to the preparation of acorn-based bread and baked goods in each of the surveyed countries. Moreover, the findings explore the production and consumption trends for these products to shed light on the possible reasons underpinning their continued use or abandonment.

The surveyed countries are grouped into two macro-regions (Mediterranean: Algeria, Italy and Syria; Central Asia and Middle East: Afghanistan, Iran and Iraq) and reported in alphabetical order.

### 4.1. Mediterranean Countries

#### 4.1.1. Algeria

Field observations and interviews in four western Algerian provinces (Tiaret, Tissemsilt, Chlef and Relizane) revealed the use of acorn flour for the production of a flatbread, locally known as *khobz el ballout*, consisting of a mixture of acorn flour, water, cereal and/or legume flour and, occasionally, yeast.

For its preparation, acorns from old trees of *Quercus ilex* subsp. *ballota* (Desf.) Samp. (=*Quercus rotundifolia* Lam.) were harvested at the end of autumn, selected (i.e., they were put into water, and only the ones that sank to the bottom were used) and boiled to facilitate the removal of the shell and the thin layer of skin enveloping the seed. Peeled seeds were boiled a second time and left in the water to remove the bitter taste. When the water turned brown (due to the expulsion of tannins), it was discarded and replaced with a new pot of boiling water. This process was repeated until the water remained clear. Leached seeds were then sun-dried or dehydrated in a traditional oven for at least half a day. They were eventually ground in cereal mills, usually the same as those used for the production of wheat flour.

According to informants, the consumption of acorn bread reached its peak during the colonial period, especially during the Algerian war (1954–1962), when wheat was scarce due to the increase in the export of national harvests to Europe by the French authorities. In that period, acorn bread was a staple food among Algerian communities living in the mountains. The *Maquis* (resistance fighters) used to prepare and eat this bread as a replacement for wheat-based bread.

Due to the availability of wheat and the better taste of wheat bread, people have now abandoned the preparation of acorn bread.

#### 4.1.2. Italy–Calabria

The exploratory fieldwork in the Catanzaro, Cosenza and Reggio Calabria provinces confirmed the past use of acorns for edible purposes. However, it highlighted the marginal role that acorn-based foods had in the local diet. Acorns of *Quercus virgiliana* (Ten.) Ten. (=*Quercus pubescens* Willd. subsp. *pubescens*) have traditionally been processed into flour that was primarily intended as feed for livestock (pigs and poultry). However, during times of famine, acorn flour was mixed with cereal and legume flour and used for the preparation of substitutes for wheat bread.

Fruits (*rugulu* as they are called in the local dialect of Cardeto village) were harvested, selected (i.e., only sealed brown and ripe fruits were used) and dried to prepare acorn flour. To this end, they were either sun-dried for some weeks, lightly roasted in a pan, slowly dried in the oven (usually the same oven used for baking bread) or a combination thereof. A 79-year-old man from Chiaravalle Centrale village described the process that his family used for drying acorns. First, acorns were sterilised by gathering the fruits into a pile and heating them inside chestnut wood containers called *ruváci*. The germinated fruits were dehydrated in the oven and subsequently stored. Dried acorns were peeled (i.e., the shell was removed) and ground into flour. The seeds were milled with cereals (e.g., maize and wheat) or, after milling, the flour was mixed with lupin (*Lupinus albus* L.), maize or oat (*Avena sativa* L.) to improve the nutritional quality and reduce the ‘sour’ taste of the acorns.

While the majority of informants remembered the production of acorn flour in detail, only the 79-year-old man from the Chiaravalle Centrale village described how to prepare acorn bread. Because the dough was soft and almost liquid, they used to place it on a cabbage (*Brassica oleracea* L.) leaf or on approximately eight chestnut (*Castanea sativa* Mill.) leaves on which an *iunta* (portion) of dough was distributed before putting it in the oven. Informants did not mention the cooking equipment or cooking times for the bread, as none of them had ever prepared it.

Even in the past, the use of acorn flour for baking purposes was very sporadic. As reported by a 70-year-old woman from the village of Cardeto, acorns were sporadically used for human consumption (as a substitute for coffee) until the early decades of the twentieth century, mainly during times of war. Thus, acorn flour was used only when no other foods were available, mainly by those families who did not grow wheat or other cereals. Chestnut flour or wheat bran mixed with lupin or grass pea (*Lathyrus sativus* L.) flour was preferred over acorn flour.

#### 4.1.3. Italy–Sardinia

Fieldwork conducted in the villages Baunei, Talana and Urzulei revealed that informants retain the memory of a long cooking process in which acorn seeds were boiled along with clay and, in some villages, ash. From this process, two different products were obtained: *lande a perra* (boiled acorns) and *lande a fitta* (the dried broth remaining after the cooking process).

For this preparation, sweet acorns (most likely of the species *Quercus ilex* L. subsp. *ilex*, *Quercus suber* L. and *Quercus pubescens* Willd. subsp. *pubescens*) were gathered between October and November on the high plateaux well-exposed to sunlight. Fruits were roasted directly on the fire, in the oven or alternatively dried on a *graticci* (trellis) to reduce moisture and facilitate the removal of the shell and skin. A 95-year-old woman from Baunei called this process *arridare*, while in Urzulei, it was known as *ispellizzare*. Once dried, the fruits were put in a linen bag (*sa berthula* or *su canissu* as it was called in Talana) and beaten on the floor.

Red clay (*torco*) was collected and mixed with water until the impurities were removed from the clay to cook the seeds. The mixture was then sieved and transferred to a copper pot (*apiolu* or *su cardaggiu*), where the seeds were added and cooked for up to eight hours while continuously stirring. The seeds were put in the boiling mixture or added to the cold liquid. Some informants also used to add ash (from the wood of oak trees) to speed up the cooking process. Once cooked, the seeds were removed from the broth, placed on a surface made of cork and left to dry.

**Figure 2:**
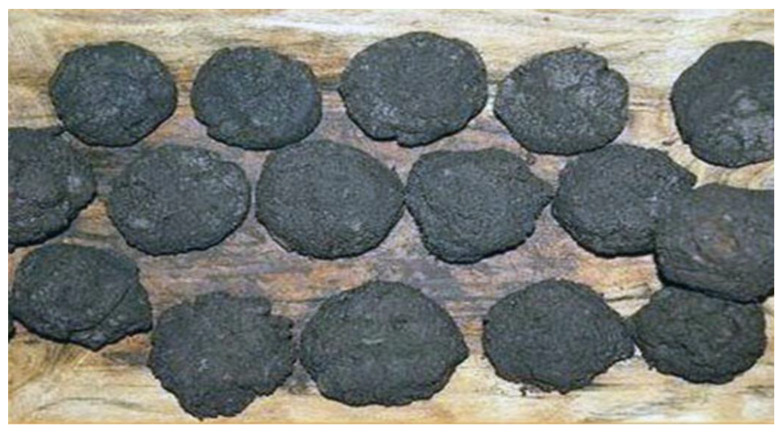
*Lande a fitta*: dried broth remaining after the cooking of acorn seeds with clay (File credits: https://www.balanofagia.org/index.php/it/la-storia/il-pane-sardo, accessed on 20 September 2022).

*Lande a perra* (cooked seeds) had a dark colour, firm texture and slightly bitter taste. According to our informants, clay (along with the drying process) partially removed the bitter taste of acorn seeds and reduced the breakage of the fruits during the cooking process. This product was mostly intended for consumption by herders during transhumance and was eaten with dairy products, such as *suru* (whey), ricotta and milk. *Lande a fitta* was similar to a small dark dry porridge with a slightly sweet and sour taste, as well as a chocolate-like texture (Figure 2). It was consumed as a pastry by children and also as a substitute for bread, especially during periods of wheat scarcity.

*Lande a perra* and *lande a fitta* had been prepared consistently until the end of World War II when, due to the shortage of cereals caused by the policies promoted by the fascist regime, it replaced wheat-based products. *Lande* was preferred to barley (*Hordeum vulgare* L.) bread, as it was considered nutritious and beneficial to digestion and was sold at a higher price than other wheat bread substitutes. However, all informants agreed that *lande* was prepared only when other foods were not available.

Although our informants still had extensive knowledge of the preparation of *lande*, currently, there are only a few people that still prepare this product and only for demonstration purposes (Pinna, 2013). The marginalisation of *lande* in Ogliastra was due to improvements in the livelihoods of local communities and the diversification of the diet. Specifically, the abandonment of traditional agro-silvopastoral and shepherding systems, to which acorn bread heritage was intimately connected, has also negatively affected the preservation of this product. As one 91-year-old man from Baunei told us: *‘When we had other stuff we put lande aside, it was out of obligation, it wasn’t out of curiosity, it was what you did when you did not have other stuff to eat’*.

#### 4.1.4. Syria–Tartus Governorate

Field observations and interviews in the western portion of Mediterranean Syria revealed remnants of traditional knowledge related to the preparation of acorn bread. In particular, in the town of Duraykish in the Tartus Governorate, scattered information on the preparation of a flatbread, locally known as *khebz dawam or khebz ballout*, was collected from an 87-year-old woman. For its preparation, sweet acorns were collected, roasted over a fire, placed in a hemp bag and crushed to remove the shells. The seeds were then boiled for several hours and dried in the sun for two to three days. Seeds were ground with a threshing rock (i.e., a roller-like tool used for milling cereals) and sieved to obtain the flour. The flour was mixed with water, kneaded and baked alone or with a portion of dough from the previous breadmaking batch (which could help in activating dough fermentation). The resulting bread, which was thicker than cereal-based flatbreads, was eaten with dairy products, such as milk, butter, yoghurt and *shanklish*, a local cheese made with cow’s or sheep’s milk.

*Khebz dawam* was prepared almost exclusively during times of famine. Our informant claimed that she used to eat this bread in her childhood (i.e., in the mid-1940s). Recalling the memory of her mother, she also claimed that acorn bread was commonly prepared in the time of *seferberlik* (wartime mobilisation during World War I) during the Ottoman Empire when wheat and barley were appropriated by the Ottoman army. This practice was abandoned in the 1950s when the availability of wheat and barley started increasing. Similarly, a 94-year-old man remarked on the importance of acorns in historical times, claiming that during the era of Ottoman occupation, when hunger was most severe, acorn-based products ‘*kept people alive during wartime*’.

### 4.2. The Middle East and Central Asia

#### 4.2.1. Afghanistan–Kunar, Nuristan, Nangarhar, Laghman, Paktia, Paktika, Ghazni and Ghor Provinces

Face-to-face and phone call interviews with 41 members of the Afghan diaspora in Pakistan (migrants coming from the provinces of Baghlan, Kunar, Kunduz, Laghman, Logar, Nangarhar and Paktia) provided evidence of the occasional use of acorns for alimentary purposes and especially of acorns roasted in hot ashes. However, in these areas, informants did not mention any use of acorn seeds or acorn flour for the production of bread.

Some evidence for acorn flour-based products was recorded, however, among the local communities who live in the forests of the Eastern parts of Afghanistan (the provinces of Kunar, Nuristan, Paktia, Paktika and some parts of Ghazni) and in Ghor province. Acorns of the species *Quercus dentata* Thunb. and *Quercus semecarpifolia Sm.* were harvested, roasted (until the shells became dark brown and the moisture evaporated) and crushed to remove the shells. Roasted sweet acorn seeds (*Quercus dentata*) were pounded with a stone mortar and turned into flour. Bitter acorn seeds (*Quercus semecarpifolia*) were, instead, boiled for a few hours and kept in water for 24 h. The water was then discarded, and the seeds were boiled again to eliminate tannins. This process was repeated up to three times, and finally, the seeds were dried in the sun. Leached seeds were pounded while adding a little water to the stone mortar; the liquid was then drained, and the flour was dried and pounded into powder.

The soft and liquid dough (made mostly with sweet acorns) was made into a thin layer and cooked in a hot pan. In the Ghor province, fat or oil was spread on the surface of the dough before cooking. The cooked bread, known as *pragi* or *nan-e-bloot,* was flat, smaller than *chapati* and had a sweet taste. It was customarily eaten with rice (*Oryza sativa* L.), meat and honey and accompanied by water or tea.

As observed during fieldwork, acorn bread played a crucial role in the food security of rural communities, and it was also consumed during periods of drought between 1996 and 2001. In some eastern rural villages of Afghanistan, *pragi* is still eaten occasionally as a medicinal food.

#### 4.2.2. Iran–Kohgiluyeh, Boyer-Ahmad, Lorestan and Kurdistan and Khuzestan Provinces

**Figure 3:**
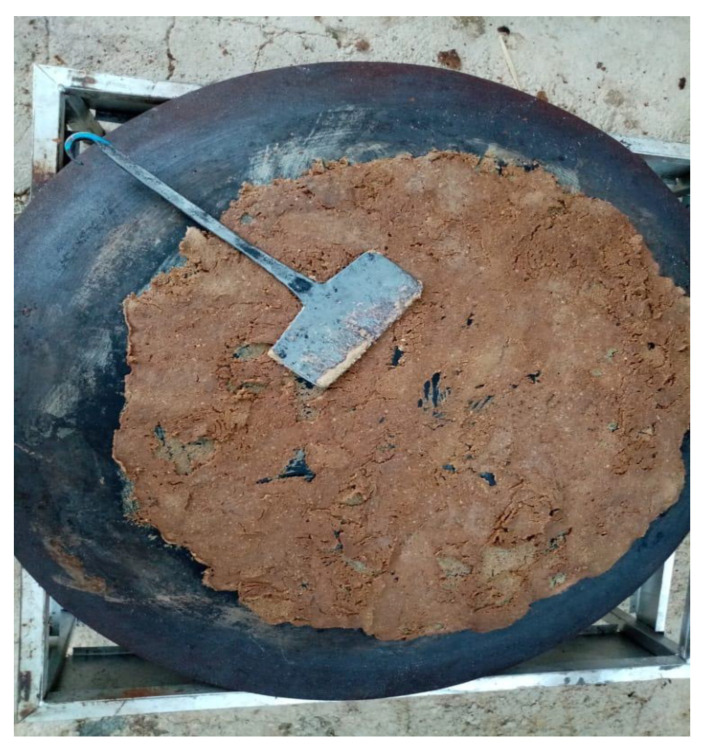
*Kalg or kezke*: acorn flatbread cooked on a convex metal griddle called a *saaj* (photo: Seyed Hamzeh Hosseini).

Field activities and observations carried out in three Iranian provinces revealed the persistence and conservation of traditional gastronomic knowledge linked to the preparation of acorn bread. In the Kohgiluyeh, Boyer-Ahmad and Lorestan provinces, acorns collected in early autumn were placed in a chamber (similar to a furnace) built with stone walls and covered with wood to dry and to facilitate the removal of the shells *(jaft*). The fruits were then beaten on a stone slab with a stick to remove the skin. Subsequently, the seeds, placed inside a container, were sprinkled with a few handfuls of roasted acorn flour (*patina*) and placed in a water stream (*shiver*). They were kept in this condition for several days to ‘sweat’ (community elders used to say that the fruits had a ‘fever’). Leached seeds were then sun-dried, ground and made into flour. The flour was kneaded and baked in a pan called a *saj*. This flatbread, locally known as *kalg* or *kezke*, had a black colour and a slightly hard consistency (Figure 3). It was traditionally eaten with meat products (broth, sheep head and kebab), wild vegetables (*Pistacia atlantica* Desf., *Gundelia tournefortii* L.) and dairy products (yoghurt, curd and butter).

**Figure 4:**
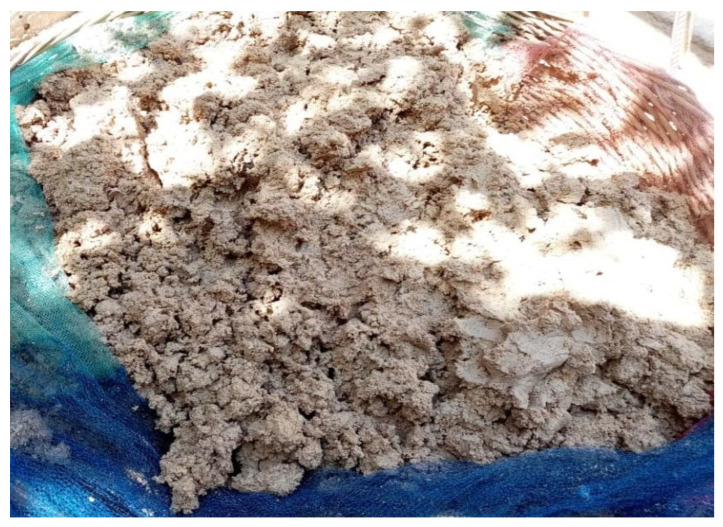
Acorn flour was mixed with warm water and made into a paste. The flour was then rinsed to remove the bitter compounds (photo: Seyed Hamzeh Hosseini).

In Iranian Kurdistan, sweet acorns (preferably from the species *Quercus brantii* Lindl., *Quercus infectoria* G.Olivier and *Quercus libani* G.Olivier) were used for the preparation of a bread called *nane belu*. The skin was removed by sun-drying the fruits and by stamping on them with the feet or by hitting them with a wooden tool called a *gelarco* while in a bag. The peeled seeds were placed on a cloth or tray and dried under the sun for at least three days to remove the excess moisture. During this time, they were stirred several times to loosen and separate the seed coat (*jaft*). The dried seeds were ground into coarse flour with a manual stone mill called a *makina*. The flour was mixed with warm water, made into a paste, placed in a bowl and covered with a thick cloth-like blanket to keep it warm (Figure 4). The flour was then rinsed with a device called a *nane shan* to remove the bitter compounds. The resulting flour was crushed again with a stone mill.

**Figure 5:**
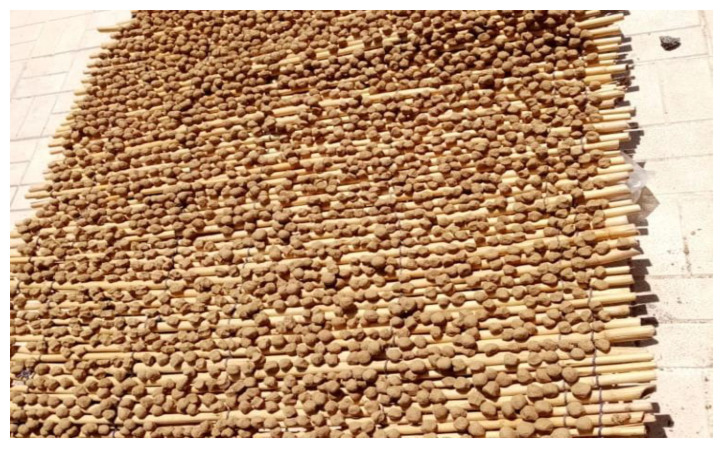
Dough made with acorn flour and water (photo: Seyed Hamzeh Hosseini).

The flour was mixed with water and moulded by hand with a tool called a *penne* to prepare the bread (Figure 5). The dough (*ganke*) was flattened and cooked on both sides on a convex metal griddle called a *saaj* [85]. The resulting bread (*kalg* or *kezke*) was coarse, dark brown and almost the size of a palm. It was usually served with dairy products (butter, yoghurt or buttermilk curd), wild herbs (e.g., *Ranunculus kochii* Ledeb., *Allium giganteum* Regel, *Eremurus spectabilis* M.Bieb., *Portulaca oleracea* L.), *doshab* (juice obtained from the fruits of *Morus nigra* L.) and in traditional dishes such as *dokheh wa* (buttermilk, rice and vegetables), *shorbau* (soup made of rice and vegetables), *doina* (condensed boiled buttermilk and rice) and *trit* (crushed bread in buttermilk).

**Figure 6:**
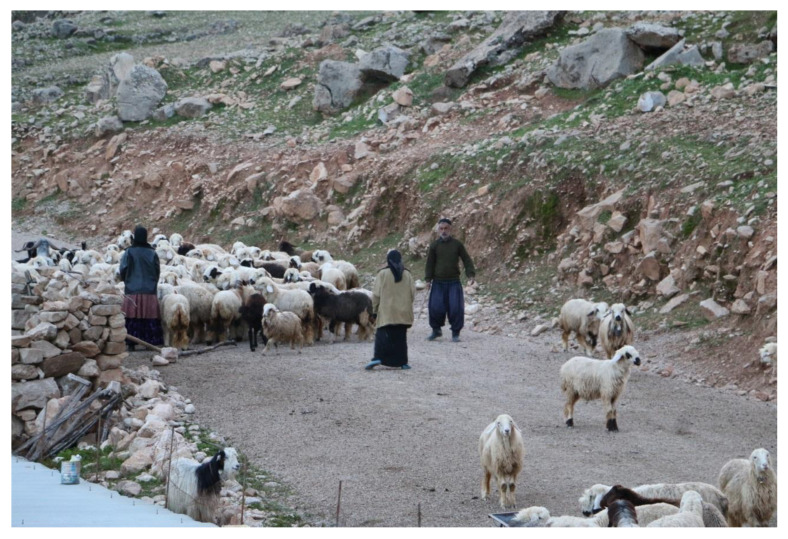
Bakhtiari shepherds in their winter pastures near the city of Masjed-Soleiman, Khuzestan province, Iran (photo: Datis Mohammadi).

In the surveyed areas, acorn bread was a staple food until about 70–80 years ago, especially during periods of famine in remote villages and nomadic areas without access to other substitutes for wheat flour. At present, acorn bread has lost importance in the local diet. One of the reasons for not using acorn bread is the easy access to wheat flour, although the long preparation process and high production cost of acorn flour have also played a role. However, in some remote rural villages in Lorestan, shepherds from the Bakhtiari tribe still rely on acorns and acorn-based products for their subsistence and medicine (Figure 6); indeed, Bakhtiari from Khuzestan used to exclusively utilize acorn flour to make bread, the preparation of which took much effort, and so it is nowadays far more convenient to buy wheat or barley flour. Two Bakhtiari study participants still retained very detailed knowledge of acorn bread; one of them was an older woman who described acorn bread preparation as follows:

*‘In years of hardship, like dry years or very cold years when people were suffering more than usual, acorn and wheat, especially acorn, had very deep roots in our culture, to feed the livestock and the men. There are several products made of acorns. I mean our acorns, have you seen our acorns? …At the end of autumn, after the first rain—if it doesn’t rain they are bitter—but after the first rain hits the trees and the fruits become sweeter, they will fall on their own. Sometimes we would pick them but usually they would fall on their own…They would bring them home, roast them, take off the shells and store them…in this way they wouldn’t go bad…Then we reached the real kernel; the kernel would be milled into flour, but not a fine flour, with something called dastar, an egg-shaped stone around 50 kg would be put on a flat stone and the kernel would become small like wheat. It would still contain bitterness… the kernels were then put in big bags of 30 or 40 kilos and put in a stream or a river for days. Then they were dried again, milled and made into bread, called kalg.’*

#### 4.2.3. Iraq–Kurdistan

Exploratory fieldwork in the eastern and southern parts of Iraqi Kurdistan showed great diversity and variability in the use of acorns for the preparation of baked goods. Flour made from sweet acorns, presumably *Quercus aegilops* (=*Quercus cerris* L.), was used for the preparation of a flatbread called *nani baru* in Bingrd and Balkha, and *astuk* in Shanidar, Barzan and Hawraman.

Acorns, harvested in autumn and wintertime, were pounded with a hammer or a stone to remove the hard coat (pericarp) of the fruits. Seeds were placed on the roof of the house to dry or were dried by roasting them in a pan at low heat. They were then boiled to eliminate the bitter taste and remove the seed coat. Finally, they were dried under the sun or on an open fire. Leached and dried seeds were processed into flour using *dastar/juni* (i.e., two large stones that grind the seeds by circulating one over the other) or *sndol/daskawan* (i.e., mortar and pestle).

Different variations of acorn bread recipes were documented during fieldwork. Overall, our informants, except for a 76-year-old man living in Hawraman, claimed to mix acorn flour with barley or wheat flour to ease the preparation of the dough (to make it more elastic), as well as to improve its taste and texture (to reduce the bitterness of the acorn flour). For the same reason, informants added other ingredients to the dough, such as salt, yeast, honey or dried mulberries. The dough was prepared by mixing water, acorn flour and other ingredients. The resulting mixture was divided into dough balls (*gunk*), flattened by hand or with a wooden roller and cooked in a *tandoor/tandwr* oven (i.e., a traditional oven made of clay) [85]. Animal fat (*kara*) was spread on the surface of the bread before cooking. The final product was smaller in size and thicker than traditional flatbread (*nan*) and has a slightly bitter taste. In Bingrd, acorn bread was traditionally eaten with dairy products, such as a drink called *dow* and/or *mastaw* (a mixture of yoghurt and water), and vegetables, such as tomatoes, young onions and parsley.

Our informants recalled the use of acorn flour during World War I, a time when people could not prepare bread with wheat. Community elders also said that, in the village of Shanidar, acorn seeds (eaten as a snack) and acorn bread were important foods between 1920 and 1945, especially for poorer community members. Starvation was the main reason why people ate acorns and used acorn flour. Nowadays, as stated by our informants, it is very rare for people to make this kind of bread, as wheat flour is much more readily available and distributed regularly by the government. While acorn bread is no longer part of the present dietary regimen of Kurdish people, its preparation is still remembered by a portion of the elders of rural villages, and it is still occasionally made during special occasions and celebrations.

## 5. Discussion

### 5.1. Acorn Bread in the Mediterranean and the Middle East: A Comparative Analysis

Table 3 summarises the main results of this study, highlighting the main similarities and differences in the preparation of acorn bread and acorn-basedproducts in the two cultural and geographical macro-regions of the Mediterranean basin, Central Asia and the Middle East.

In the following sections, a comparative analysis regarding the ingredients and preparation of these products, as well as the dynamics underpinning the conservation or erosion of the traditional gastronomic knowledge associated with these practices, is presented.

#### 5.1.1. Ingredients, Preparation Techniques and Consumption Practices

Overall, our informants showed a preference for the use of sweet varieties of acorns for the preparation of acorn bread and acorn-based baked goods. In particular, as observed in Italy, Syria, Afghanistan, Iran and Iraq, whenever available, study participants preferred sweet varieties (i.e., those fruits with a lower tannin content) over bitter ones. According to our informants, the sweet varieties in the Mediterranean were *Quercus ilex* subsp. *ilex.*, *Quercus suber*, and *Quercus pubescens* subsp. *pubescens*, while, in Central Asia and the Middle East, study participants mentioned *Quercus dentata*, *Quercus brantii*, *Quercus infectoria*, *Quercus libani* and *Quercus aegilops* as the most commonly used species for the preparation of acorn flour and acorn bread. While the preference for sweet varieties of acorns in the Mediterranean region had already been reported in historical sources [41] and in previous studies [28,57,64], our study outlined a similar situation in Central Asia and the Middle East.

The study showed a great distinction in acorn-based bread in the two macro-regions, especially in terms of ingredients, production of flour and physical characteristics of the cooked products. In Mediterranean regions, acorn seeds were traditionally processed into flour and used for breadmaking in its pure form, as in Syria, or, as in Algeria and southern Italy, mixed with gluten-containing cereal flour to improve the rheological properties of dough or with legume flours. In Central Asia and the Middle East, acorn flour was used for the preparation of flatbread, usually by mixing it with cereal flour and sometimes by adding other ingredients to mitigate the bitter taste and astringency of the acorn seeds. These undesired sensory characteristics are due to tannins, water-soluble antinutritional factors able to form chemical complexes principally with proteins (limiting their digestibility) but also with polysaccharides [86,87]. The binding of salivary proline-rich proteins by tannins causes their precipitation and results in astringency [88].

In both macro-regions, to reduce tannin concentration, acorn seeds, either whole or in the form of a coarse meal, were subjected to a leaching process. Depending on the area and the acorn varieties used, this process (the number of steps and duration of which seems to be linked to the concentration of bitter compounds in the fruits) involved roasting (Italy–Calabria), boiling (Algeria, Afghanistan, Iraq and Syria), or soaking the acorns in cold water (Iran), and subsequently drying the seeds. Although in less detail, similar practices have been documented in previous studies on the alimentary use of acorns by Younker [27] and Potts [35] in Iran, as well as by Mason and Nesbit [33] in southeast Turkey. However, to our knowledge, no detailed documentation on the debittering and leaching processes in Algeria and Syria has been carried out thus far.

A different leaching technique has been recorded in the Ogliastra region, Sardinia. As already documented by Usai [65] and Pinna [68], acorn seeds were processed into a dried porridge with unique techniques and additives, namely the use of iron-rich clay and ash to reduce the bitterness of the seeds. Similar products and detoxification techniques were documented in North America among the Pomo Indians of California [30] and other Native American communities [7,69].

Regarding the cooking process, in the Mediterranean, loaves of bread were usually baked in a wooden oven. In Central Asia and the Middle East, the dough was flattened and cooked on a traditional baking griddle (*saj*), as in Afghanistan and Iran, or in a vertical oven (*tandoor*), as in Iraqi Kurdistan. These cooking tools are used for the baking of unleavened bread, which are a traditional element of the food heritage of rural communities inhabiting these regions [85]. As observed in Iran, similar acorn-based products (*kalg*) and associated processing and consumption practices (i.e., with dairy products such as yoghurt and curd), which were documented in the second half of the nineteenth and the twentieth centuries (see the work of Potts [35] on balanophagy in Iran), are still part of the local gastronomy and diet.

While information and memories regarding the leaching of acorns and their processing into flour are still rich and detailed, our informants described their preparation phases (i.e., kneading techniques, quantities of ingredients, fermentation conditions and cooking times) in scant detail (or not at all). In this regard, fermentation was particularly neglected and was nearly not reported by informants from the investigated areas; however, it is possible they did not describe the details of this processing phase because they were not personally knowledgeable in the preparation of acorn bread. Fermentation is a kind of ‘hidden’ process that may remain less evident and is linked to empirical experience, which was lacking in many of the informants. 

The erosion of the traditional knowledge associated with the preparation of acorn-based products was more evident in the Mediterranean countries since, as already observed elsewhere [28,68], these foods are sporadically used and marginalised in the local diets. At least until the first half of the twentieth century, acorn-based products were, in both macro-regions, an integral element of the food basket of pastoral and agro-pastoral communities in times of scarcity, especially among poorer people who had difficulty accessing cereals. Acorns in the form of bread replaced cereal-based bread (wheat bread above all), and acorn bread was consumed with food products linked to pastoral foodscapes, especially dairy products, meat-based dishes (e.g., sheep and goat meat and offal) and wild vegetables.

Although the gastronomic use of acorns has almost completely disappeared in Mediterranean countries, our study documented evidence of the modern continuation of this practice in several of the surveyed countries in the Middle East and Central Asia, especially in Iran and Afghanistan. Overall, in terms of processing, consumption and reasons for use, our research showed clear interregional parallels, while a degree of overall continuity may be observed with historical and archaeological practices in these areas.

#### 5.1.2. Alimentary Role and Associated Values

Although informants in almost all of the surveyed areas acknowledged the past role of acorn-based bread as a safety net during periods of starvation, they remarked on its unpleasant taste, perceived scarce nutritional value and long and difficult preparation, as well as the association of this product with times of poverty. As recalled by an informant in the Afghan province of Ghor, this product did not have ‘*the best taste*’, but when no other food was available, ‘*it tasted as good as wheat or cornbread*.’ Another informant in Afghanistan remembered his grandfather saying that, in times of famine, he needed to grind acorns into flour, thus showing the poor gastronomic consideration of this product. In Syria, acorn bread was regarded as ‘*not of high nutritional value*’ but was also recognised as crucial in ‘*keeping people alive during wartime*.’ Moreover, in the Iraqi village of Balkha, starvation was identified as the main reason for the preparation and consumption of acorn bread.

Our findings, therefore, support the hypothesis that acorn bread, at least during the last two centuries, has mostly been used as a famine food. However, the role and value attributed to acorns vary according to the geographical conditions and socioeconomic characteristics of the surveyed regions. In this regard, the perception of the alimentary value of acorns was higher among communities that based their livelihoods on the exploitation of pastoral systems in the Middle East and Central Asia [27,89] and Mediterranean agro-silvopastoral systems [1,90], i.e., where cereal cultivation was difficult and, in those areas, where oak forests predominated over other tree species.

The relevance of acorns and acorn bread (*lande*) in Ogliastra (Sardinia, Italy) was, for instance, strongly connected to the role that oak trees have played in the traditional agro-silvopastoral livelihoods of local herders (e.g., as a source of wood and fodder for pigs and sheep). This is different from other Italian regions, where chestnut played a prominent role in the agro-silvopastoral systems and foodscape. The limited presence of chestnut trees and, more generally, the scarcity of food resources in the Ogliastra area could have had a central role in the development of the traditional ecological and gastronomic knowledge linked to the management and use of oak trees and acorns. The importance of oak trees is also illustrated by the regulations regarding the management of *ghiandatico* (use of the acorn forest), that is, still part of the usi civici (civic uses) in different villages of Alta Ogliastra. Conversely, the predominance of chestnut forests in Calabria (southern Italy), alongside the chestnut’s greater palatability, the ease of processing its fruits and the greater availability of cereals might have favoured the use of acorns in the local diet andgastronomy.

A similar trend may be observed in other surveyed areas (most notably in Syria, Iran, Iraq and Afghanistan). The ecological abundance of oak trees, as well as the crucial cultural values attributed to them (i.e., the collection of ‘Kurdish manna’ from unripe acorns in June or the use of acorns in traditional medicine [91]), could have fostered its consumption and utilisation as emergency food during times of scarcity or, as stated by Rosenberg [12] and Potts [35], as a staple food in those areas where the cultivation of cereals was extremely difficult because of poor water availability, geomorphological features of the territory and other similar factors. Especially in Kurdistan, the key role of acorns in the local culture may have positively shaped the perceived values and narratives related to this product; we documented that one study participant in the village of Yasuj referred to the acorn as the ‘holy fruit’.

#### 5.1.3. Main Drivers behind the Continuation/Abandonment of Acorn Bread Preparation and Consumption

As also recorded elsewhere [27,28,57], this study confirmed that the consumption of acorn bread was mostly restricted to times of famine and scarcity. Both in the Mediterranean and the Middle East, an increase in the consumption of acorn-based products was observed during times of war (e.g., during the fascist regime in Italy, the Algerian war, World War I and World War II in Iran and Iraq, as well as during *seferberlik* in Syria) as a consequence of disruptions in the production and distribution of cereals.

Despite vivid remembrance of the preparation of acorn flour and bread, the evidence gathered during fieldwork showed that, in the last five decades, especially in the Mediterranean basin, these products have been almost entirely abandoned. Our study thus showed a similarity in the reasons behind the erosion of this practice throughout the two macro-regions. In almost all of the areas investigated, the use of acorns and acorn flour has been progressively decreasing due to an increase in the availability and affordability of wheat and other grains, improved socioeconomic conditions and lifestyle transformations. Moreover, acorn bread is no longer produced due to the difficulty of processing acorn seeds (both because it is a time-consuming activity and due to the loss of knowledge regarding the processing of acorn seeds), as well as its poor palatability.

The interviewees claimed to have prepared and eaten this bread up through the first half of the twentieth century. Except for Iran and Afghanistan, where acorn bread was used as a staple during the 1996–2001 droughts, none of them had prepared acorn bread in the last 50–60 years apart from demonstrations and occasional celebrations (Afghanistan, Iran and Sardinia) or medicinal uses, as in Afghanistan and Iranian Kurdistan.

### 5.2. Beyond Acorn Bread: From Abandonment to Evolution

Interest in acorns as a food has gained considerable momentum in recent years. Researchers have carried out studies on the nutritional and phytochemical profile of acorns and their related health effects, thus arousing strong interest in exploring new applications in various fields. The high potential of acorns for use in human nutrition has been confirmed by the proposed use of acorns in conventional and gluten-free biscuits [37,92], conventional and gluten-free bread [93,94,95,96,97], cakes [98], noodles [99], coffee substitutes [100] and oil [23,101], not to mention their possible biotechnological use in pharmaceutics and biomaterials [102,103,104]. The production of bread, in particular, has been revived by using acorn flour mixed with other flours and fermenting with specific strains of lactic acid bacteria (LAB) to decrease the phytic acid content and increase the number of phenolic compounds (from tannins), which in turn, enhances antioxidant activity [95,96]. Acorns can also represent the raw material for the production of food ingredients to be used in nutraceuticals, food supplements and functional foods [105]. In this regard, the extraction of bioactive compounds such as phytosterols, tocopherols, carotenoids and phenolics [106], as well as the recovery of proteins [107] and starch [108], have been proposed.

Although their composition varies from species to species, acorns are rich in nutrients, such as carbohydrates and fats (mainly unsaturated), with moderate amounts of proteins and high levels of calcium, iron, magnesium, phosphorus, potassium, vitamins A and E [29,36,109,110], all of which contribute to good nutritional status. In addition, the number of consumers who are reducing their intake of animal-based foods is increasing globally, resulting in a growing market for plant-based products, and acorn-derived foods fit well with this trend. Moreover, a recent study has shown the economic feasibility of creating an acorn supply chain for food production [111].

Traditionally acorns were an emergency or ‘bailout’ crop for when others failed or were unavailable, yet today, acorns may be able to help us face new challenges. They can, for instance, play a role in the diversification of food sources and add an additional element of climatic resilience, helping to ensure food security [112]. The renewed use of acorns could also contribute to the maintenance of rural and marginal areas exposed to the risk of degradation deriving from anthropic activities. Therefore, the reintroduction of this underexploited but highly nutritious crop into the food system by adequately innovating acorn-derived foods through the implementation of modern food technologies and going beyond the preparation of acorn bread could meet the demand of modern consumers for healthier foods and could be a response to future food security concerns at the same time.

### 5.3. Acorns as a Potential Resource to Foster Food Security, Rural Development and Innovation

Our study showed that acorn bread appears to be a distinctive element of local food heritage in many areas in the Mediterranean, Near East and Central Asia, but also one that is on the verge of extinction. In the face of this situation, the documentation of traditional gastronomic knowledge related to acorn and acorn-based products could represent a valuable resource for the future of rural and marginal regions for different and often interconnected reasons.

First, taking into account the nutritional value of acorn seeds [29,36], the findings of the present study may represent a valuable source of baseline data for the implementation of context-based interventions aimed at promoting the alimentary use of acorns and, in doing so, reinforcing the food security of rural communities in the studied areas. Moreover, considering the role that acorn-based products had during times of famine and food shortages, the recovery of knowledge related to the management and uses of *Quercus* wood and acorn-based products could positively impact the resilience capacity of local communities in the future. In this regard, keeping alive and reinforcing the memory of acorn processing could possibly have great value in the context of climate change and nutritional intolerances, in which alternatives to cereals are increasingly needed.

Secondly, acorn bread, alongside other culinary uses of acorns such as we observed in historical sources, may represent a new source of inspiration and an item to be included in new menus or dishes. Furthermore, acknowledging the emerging trends and the numerous and varied potential applications of acorns in the food sector [23,37,93,94,95,96,97,98,99,100,101], the findings of this study may represent a valuable resource for the development of innovative products, appreciated by modern consumers unfamiliar with the rural lifestyle and the acorn’s past role as a famine food, but who are increasingly aware of the richness in macro- and micronutrients of these fruits.

Thirdly, in the context of intensifying food tourism and growing demand for local speciality foods and cuisines [113], acorn bread shows potential in terms of local characterisation, adding a new dimension to its use by communities that maintain this food tradition and heritage. In this respect, in the case of Ogliastra, this process seems to be more advanced, and the product appears to be an integral part of the narrative of the area (Bondioli, personal observation). Even more than the product itself, the complex and labour-intensive process that the transformation of acorns requires can be seen as a source of inspiration for experiential activities. Thus, as already stated, the valorisation of acorns and acorn foodscapes could represent a potentially crucial resource for the re-activation of the local economies of rural and marginal areas [111,114].

## 6. Conclusions

This study contributes to the scarce literature on traditional gastronomic knowledge associated with the production of acorn-based bread. By comparing the ethnographic data from select countries, our analysis highlighted, in particular, distinct trajectories in the development of acorn-based bread in the Mediterranean basin, Central Asia and the Middle East. In so doing, it highlighted some differences in terms of ingredients, processing techniques and baking methods. In Central Asia and the Middle East, acorn seeds, processed through debittering and leaching techniques, were used in the form of flour for the production of flatbreads that were cooked on a baking griddle and in a vertical oven. In the Mediterranean (except for Sardinia), they were roasted, dried and ground into flour, usually mixed with cereal flour and baked in a dome-shaped oven. Despite these differences, acorn bread appears to be primarily an emergency food resource used by pastoral and agro-pastoral communities when facing limited access to cereals. The complex processing and treatment that acorn seeds require for debittering prior to breadmaking particularly explains the nature of this product as an emergency bread substitute. Its status was confirmed by the progressive marginalisation of this product from the everyday diet as a result of the economic and social transformations that have occurred over the past few decades.

Our study acknowledges the cultural importance of acorn fruits and acorn-based products, as well as their potentially crucial applications as an alternative solution for the future of rural economies. Our results suggest that the recovery of acorn-based products and associated traditional knowledge may foster the sustainable development of rural and marginal regions in the Mediterranean, Central Asia and the Middle East. This could help to reinforce the resilience of local communities and thus increase food security. Furthermore, reassessing acorns as a foodstuff may aid in developing innovative products in line with emerging trends in the food sector, which is looking for new non-cereal-based bakery products and other novel culinary applications.

Despite the fragmentary nature of the ethnographic resources used, this study revealed the wide geographical area in which acorn-based bread items were made and the different processing methods used. Future research, potentially based on a more standardised form of data collection, may add to the description and assessment of the practices and knowledge underlying the preparation of these products. Furthermore, more attention could be paid to the variation and diversity in the use of the fruits of different *Quercus* species (e.g., perennial versus deciduous trees) for the preparation of acorn-based products. At the same time, further research should be conducted to enrich our understanding of acorn consumption as a gastronomic phenomenon and the cultural values attached to it, as well as to explore ad-hoc strategies to support the promotion of these products. Lastly, future research may involve the exploration of oak genetic variability to identify sweeter varieties and easier detoxification techniques, as well as the exploration of fermentation practices using selected tannin-tolerant starters that could make the use of acorns more popular, thereby combining old traditions and new scientific knowledge.

## Figures and Tables

**Table 1:** Sociodemographic characteristics of the informants.

Interviewer(s)	Country	Region(s) or Province(s)	Village(s)	Number of Informants	Sex	Age
A.F.	Afghanistan	Kunar, Nuristan, Nangarhar, Laghman, Paktia, Paktika, Ghazni	Different villages	6	M	50, 54, 62, 65, 75, 77
		Ghor	Tar Būlāq, Taywara, Sagar	4	M	48, 52, 66, 67
M.M.D	Algeria	Chlef	Chlef	5	F	96
		Relizane	Relizane		F	80
		Tiaret	Tiaret		M	82
					F	92
		Tissemsilt	Tissemsilt		M	76
H.I.H.A. and D.M.	Iran	Kohgiluyeh and Boyer-Ahmad, Lorestan, Khuzestan	Yasuj, Si sakht, Khorramabad, Ab Bid	22	M (7) F (15)	45–60 (7) 60+ (15)
		Kurdistan	Sarvabad, Marivan	9	M (3) F (6)	45–60 (3) 60+ (6)
F.H.S.H., T.F. and K.D.I.	Iraq	Kurdistan	Balkha, Hawraman, Shanidar, Bingrd, Ranya,	5	M M F F M	66 76 92 88 90
H.M.A.						
C.B.	Italy (Sardinia)	Nuoro–Ogliastra subregion	Baunei,	8	M	83, 91, 95, 95
			Talana,		M	88
			Urzulei,		F	90,92
C.M.M.	Italy (Calabria)	Reggio Calabria,	Cardeto,	6	F	48, 70, 82, 93
		Catanzaro,	Chiaravalle Centrale,		M	79
		Cosenza,	Santa Sofia D’Epiro,		M	81
C.K.	Syria	Tartus Governorate	Bahuzi,	2	M	94
N.S.			Duraykish		F	87

**Table 2:** Overview of acorn-based products reported in all case studies by country and region/province.

Country	Region (Province)/Villages	Product Category	Local Name(s) ^a^	Taxon	Ingredients	Drying and Cleaning/Shelling	Leaching/ Debittering	Flour Preparation	Breadmaking
Algeria	Tiaret, Tissemsilt, Chlef, Relizane/ Tiaret, Tissemsilt, Chlef, Relizane	Bread	*Khobz el Ballout* (A)	*Quercus rotundifolia* Lam.	Acorn Wheat flour Carob flour Water Yeast (optional)	***Cleaning/Shelling*****:** Fruits were selected and boiled, and the outer coat was removed.	***Boiling*****:** Seeds were boiled and left for a day, and the water was discarded. The process was repeated until the water remained transparent. ***Drying*****:** Boiled seeds were sun-dried or dehydrated in an oven for half a day.	***Pounding/*** ***Milling*****:** Seeds were ground in traditional mills.	***Dough*****:** Flour was mixed with water, a small portion of wheat or carob flour and, occasionally, yeast. ***Cooking*****:** The dough was baked in an oven.
Italy	Sardinia (Nuoro–Ogliastra subregion)/Baunei	Acorn meal	*Lande* (Sa)	*Quercus ilex* L. subsp. *ilex* Quercus suber L. *Quercus pubescens* Willd. subsp. *pubescens*	Acorn Clay Ash Water	***Drying*****:** Fruits were dried over the fire in a warm oven. ***Cleaning/Shelling*****:** Fruits were put in a cloth bag (*sa berthula*) and pounded to remove the shells and skin.	***Clay preparation*****:** Clay (*torco*) was harvested and sieved to remove stones and impurities. ***Boiling*****:** Peeled seeds, clay and ash were mixed with water and brought to a boil in a big copper pot (*su caddargiu*). The mixture was cooked for at least five hours.	-	***Cooked acorns*****:** Boiled seeds were removed from the broth and ready for consumption. ***Broth*****:** Once cold, the broth was put on a table made of cork skin and left to dry.
Italy	Sardinia (Nuoro–Ogliastra subregion)/Baunei	Acorn meal	*Lande a fitta* (Broth) (Sa) *Lande a perra* (Boiled seeds) (Sa)	*Quercus ilex* L. subsp. *ilex* *Quercus suber* L. *Quercus pubescens* Willd. subsp. *pubescens*	Acorn Clay Ash Water	***Cleaning/Shelling*****:** The hard coats were removed by drying (*arridare*) the fruits on trellises.	***Boiling*****:** Dried seeds were boiled in a mixture of water, clay, and ashes. Ashes were collected in an area called *sa cinisargia*, where people used to burn old or dead plants.	-	***Cooked acorns*****:** Seeds were boiled until soft and removed from the broth. Adding clay helps to reduce the breaking of fruits during cooking. ***Broth*****:** The mixture remaining in the pot was left to dry on a table made of cork (*ortigu*).
Italy	Sardinia (Nuoro–Ogliastra subregion)/Baunei	Acorn meal	*Tzipulas de lande* (Broth) (Sa)	*Quercus ilex* L. subsp. *ilex* *Quercus suber* L. *Quercus pubescens* Willd. subsp. *pubescens*	Acorn Clay Ash Water	-	***Clay preparation*****:** Red clay was harvested in the pasture and sifted with a sieve, towel or handkerchief to remove the impurities. ***Boiling*****:** Dried seeds were boiled in a mixture of water, clay, and ashes.	-	-
Italy	Sardinia (Nuoro–Ogliastra subregion)/Baunei	Acorn meal	-	*Quercus ilex* L. subsp. *ilex* *Quercus suber* L. *Quercus pubescens* Willd. subsp. *pubescens*	Acorn Clay Ash Water	***Drying*****:** Fruits were dried on the top of trellises (*graticci*) made of reeds. ***Cleaning/Shelling*****:** Fruits were put in a sack and pounded to remove the shells and skin.	***Boiling*****:** Peeled seeds were divided into halves and cooked in a mixture of water and clay. Ash (a few spoonfuls) was added to facilitate the cooking process.	-	***Cooked acorns*****:** Once cooked, the seeds were removed from the broth with a perforated spoon and put into a colander to drain. ***Broth*****:** The broth (i.e., water and acorn leftovers) was left to dry on a table made of cork and cut into slices.
Italy	Sardinia (Nuoro–Ogliastra subregion)/Baunei	Acorn meal	-	*Quercus ilex* L. subsp. *ilex* *Quercus suber* L. *Quercus pubescens* Willd. subsp. *pubescens*	Acorn Clay Ash Water	-	***Clay preparation*****:** Men harvested clay (*torco*) in the mountains. ***Boiling*****:** Seeds were boiled in a mixture of water and clay. Sometimes ash was added.	-	-
Italy	Sardinia (Nuoro–Ogliastra subregion)/Talana	Acorn meal	*Lande a perra* (Boiled acorns) (Sa)	*Quercus ilex* L. subsp. *ilex* *Quercus suber* L. *Quercus pubescens* Willd. subsp. *pubescens*	Acorn Clay Water	***Drying*****:** Acorns were harvested and hung near the fireplace. ***Cleaning/Shelling*****:** The hard coat and the skin were removed with the same techniques used for chestnuts.	***Clay preparation*****:** Clay was mixed with water, left to rest for a couple of days and sieved with a cloth (the same used for cheese making). ***Boiling*****:** The sieved mixture was boiled, and the seeds were cut into two halves and added to the boiling liquid.	-	***Cooked acorns*****:** Once cooked, they were removed from the broth with a ladle.
Italy	Sardinia (Nuoro–Ogliastra subregion)/Urzulei	Acorn meal	-	*Quercus ilex* L. subsp. *ilex* *Quercus suber* L. *Quercus pubescens* Willd. subsp. *pubescens*	Acorn Clay Water	***Cleaning/Shelling*****:** Fruits were roasted and pounded to remove the hard coat and skin.	***Clay preparation*****:** Red clay was mixed with water in a pot (*apiolu*) to separate the clay from the earth (since clay is lighter than the earth, it floats on the surface). ***Boiling*****:** Seeds were added to cold water and cooked for up to eight hours. The cooking process was performed over at least two days. The liquid was left to cool down during the night and brought back to a boil the day after.	-	***Cooked acorns*****:** Seeds were boiled until they acquired a sweet taste. They turned black as coal, and a light patina of clay remained on the surface.
Italy	Sardinia (Nuoro–Ogliastra subregion)/Urzulei	Acorn meal	*Su pan’e lande* (Broth) (Sa)	*Quercus ilex* L. subsp. *ilex* *Quercus suber* L. *Quercus pubescens* Willd. subsp. *pubescens*	Acorn Clay Water	***Drying*****:** Fruits were dried over a fire to facilitate the removal of the hard coat (*sa camisola*). ***Cleaning/Shelling*****:** Dried fruits were placed in a bag (*su cannissu*) and pounded on stones. This process was called *ispellizzare*.	***Clay preparation*****:** Clay (*torco*) was harvested from the wall of caves, dissolved in water left for a few hours and filtered. ***Boiling*****:** The mixture was brought to a boil in a copper pot, and the seeds were added and cooked for up to eight hours while continuously stirring.	-	-
Italy	Calabria (Reggio Calabria)/Cardeto	Flour	*Rugulu/Ghjjanda* (Fruit) (C)	*Quercus virgiliana* (Ten.) Ten. (=*Quercus pubescens* Willd. subsp. *pubescens*)	-	***Cleaning/Shelling*****:** Fruits were roasted to remove the hard coat and the skin.	-	***Pounding*** ***/Milling*****:** Seeds were ground, and sometimes maize, wheat or oats were added.	-
Italy	Calabria (Reggio Calabria)/Cardeto	Flour	*Rugulu/Ghjjanda* (Fruit) (C)	*Quercus virgiliana* (Ten.) Ten. (=*Quercus pubescens* Willd. subsp. *pubescens*)	-	***Drying*****:** Fruits were roasted.	-	***Pounding*** ***/Milling*****:** Dried seeds were ground with maize in a cereal mill to obtain coarse flour.	-
Italy	Calabria (Reggio Calabria)/Cardeto	Flour	-	*Quercus virgiliana* (Ten.) Ten. (=*Quercus pubescens* Willd. subsp. *pubescens*)	-	***Drying*****:** Fruits were lightly roasted or sun-dried for a few weeks to facilitate the removal of the hard coat and skin.	***Roasting*****:** Seeds were roasted until golden	***Pounding/Milling***:Roasted seeds were ground into coarse flour.	-
Italy	Calabria (Reggio Calabria)/Cardeto	Flour	-	*Quercus virgiliana* (Ten.) Ten. (=*Quercus pubescens* Willd. subsp. *pubescens*)	-	***Cleaning/*** ***Shelling*****:** Ripe fruits were crushed, and the hard coat was removed.	***Boiling*****:** Seeds were boiled in water. The water was discarded, and the operation was repeated until the liquid remained clear. They were subsequently sundried.	***Pounding/*** ***Milling***	-
Italy	Calabria (Catanzaro)/Chiaravalle Centrale	Bread	-	*Quercus virgiliana* (Ten.) Ten. (=*Quercus pubescens* Willd. subsp. *pubescens*)	Acorn flour Cereal flour Legume flour Water	***Drying*****:** Fruits were germinated inside a chestnut wood container (*ruváci*), dried in the oven, and stored in jute sacks.	-	***Pounding/Milling*****:** Dried seeds were ground and mixed with maize, oats and lupin flour.	***Dough*****:** Flour was mixed with water, grain or legumes to obtain a liquid dough. ***Cooking*****:** A portion (*iunta*) of dough was placed on cabbage or chestnut leaves and baked in the oven.
Italy	Calabria (Cosenza)/Santa Sofia D’epiro	Flour	-	*Quercus virgiliana* (Ten.) Ten. (=*Quercus pubescens* Willd. subsp. *pubescens*)	-	***Drying*****:** Fruits were dried in the oven, and the shell was removed.	-	***Pounding/Milling***: Dried seeds were ground into coarse flour.	-
Syria	Tartus Governorate/Bahuzi	Flatbread	-	Sweet acorns	-	***Drying*****:** Fruits were roasted over a fire until the shells started to break.	-	-	-
Syria	Tartus Governorate/Duraykish	Flatbread	*Khebz Dawam/Khebz Ballout* (A)	Sweet acorns	Acorn flour Water	***Cleaning/Shelling*****:** Fruits were roasted on a plate over a fire until the shells started to break. They were then placed in a small hemp bag and crushed to remove the seeds.	***Boiling*****:** The seeds were boiled for several hours. ***Drying*****:** The boiled seeds were sun-dried for 2 to 3 days.	***Pounding/Milling*****:** Dried seeds were crushed with a threshing rock and sifted.	***Dough*****:** The flour was mixed with the remaining baked flour from the last few days and kneaded. ***Cooking*****:** The dough was baked in an oven.
Afghanistan	Kunar, Nuristan, Nangarhar, Laghman, Paktia, Paktika, Ghazni/Different villages	Flatbread	*Pragi* (D)	*Quercus dentata* Thunb. *Quercus semecarpifolia* Sm.	Acorn flour Water	***Drying*****:** Fruits were roasted until the colour of the shells became dark brown and the moisture evaporated. ***Cleaning/Shelling*****:** Fruits were crushed with a stone.	***Boiling and drying*** (only for *Quercus semecarpifolia*): Seeds were boiled 2–3 times, and the water was discarded. Seeds were sundried. ***Pounding and washing***(*Quercus semecarpifolia*): Seeds were pounded, and a little water was added inside the stone mortar to wash the pounded seed	***Pounding*** (*Quercus dentata*): Seeds were made into flour with a stone mortar. (*Quercus semecarpifolia*): the mixture was dried and milled into flour.	***Dough*****:** Flour and water were kneaded for 30–40 min, and the dough was flattened into a thin layer. ***Cooking*****:** The flatbread was cooked in a hot pan.
Afghanistan	Ghor/Tar Būlāq village, Taywara and Sagar districts	Flatbread	*Nan-* *e-Bloot* (D)	*Quercus dentata* Thunb. *Quercus semecarpifolia* Sm.	Acorn flour Water	***Drying*****:** Fruits were roasted until the colour of the shells became dark brown and moisture evaporated. ***Cleaning/Shelling*****:** Fruits were crushed with a stone.	-	***Pounding*****:** Roasted seeds were made into flour.	***Dough*****:** Flour and water were kneaded to obtain a soft-liquid dough. ***Cooking*****:** The mixture was cooked in a hot pan (*tawa pan*) with some oil.
Iran	Kohgiluyeh and Boyer-Ahmad, Lorestan, and Kuzhestan/Yasuj, Si sakht, Khorramabad, Ab Bid	Flatbread	*Kalg/Kezke* (L)	*Quercus brantii* Lindl. *Quercus infectoria* G.Olivier *Quercus libani* G.Olivier	Acorn flour Water	***Cleaning/Shelling*****:** Fruits were placed in a chamber built with stone walls and covered with wood to dry and facilitate the removal of the shells (*jaft*). The fruits were beaten on a stone slab with a stick to remove the skin.	***Washing*****:** Seeds were put inside a container, sprinkled with a few handfuls of roasted acorn flour (*patina*) and placed in a water stream (*shiver*).	***Pounding/Milling*****:** Leached seeds were sun-dried, ground and turned into flour.	***Cooking*****:** The flour was kneaded with water and baked in a pan (*saj*).
Iran	Kurdistan/Sarvabad, Marivan	Flatbread	*Nane* *balu* (L)	*Quercus brantii* Lindl. *Quercus infectoria* G.Olivier *Quercus libani* G.Olivier	Acorn flour Water	***Cleaning/Shelling*****:** The skin of the fruits was removed by sun-drying the fruits and by stamping on them with their feet or by hitting them with a wooden stick (*gelarco*) while in a bag. ***Drying*****:** The fruits were placed on a cloth or tray and dried under the sun for at least three days. They were stirred several times to separate the seed coat (*jaft*).	***Pounding and washing*****:** Dried seeds were ground into coarse flour with a manual stone mill (*makina*), mixed with warm water, placed in a bowl and covered with a thick cloth-like blanket. The flour was rinsed with a device called a *nane shan* to remove the bitter compounds.	***Pounding/Milling*****:** The coarse meal was ground with a stone mill.	***Dough*****:** The flour was mixed with water and moulded by hand with a tool called a *penne*. ***Cooking*****:** The dough (*ganke*) was flattened and cooked on a convex metal griddle called a *saaj*.
Iraq	Kurdistan/Ranya	Flatbread	-	*Quercus aegilops* Scop. (=*Quercus cerris* L.)	Acorn flour Water Acorn flour Wheat or Barley flour Water Salt Yeast Sugar	***Cleaning/Shelling*****:** The hard coat of the fruit was removed with a hammer or stone, and the seeds were sun-dried. Hard coats could also be removed by roasting the fruits at low heat.	-	-	***Dough*****:** It consisted of a mixture of 80% oak flour, 20% barley or wheat flour, salt and yeast. Sugar could be added to improve the taste. The mixture was mixed with water in a small container, separated into small pieces and flattened. ***Cooking*****:** The flatbread was cooked in a *tandwr* (a tool made from clay). Vegetable oil could be added to the surface before cooking.
Iraq	Kurdistan/Bingrd	Flatbread	*Nani* *barw* (B)	*Quercus aegilops* Scop. (=*Quercus cerris* L.)	Acorn flour Barley flour Water Salt	***Cleaning/Shelling*****:** The hard coat of the seed was broken with a hammer or stone. ***Drying*****:** The seeds were sun-dried on the roof of the house for several days.	-	***Pounding/Milling*****:** Dried seeds were ground with a *dastar* (two large stones that grind the seeds by circulating one over the other) or using a mortar and pestle (*sndol*).	***Dough*****:** Flour was mixed with water, barley flour and salt. The mixture was kneaded, divided into small pieces and flattened. ***Cooking*****:** The flatbread was cooked in a *tandoor* made of clay.
Iraq	Kurdistan/Balkha, Hawraman	Flatbread	*Nani* *baru* (B)	*Quercus aegilops* Scop. (=*Quercus cerris* L.)	Acorn flour Wheat flour Water Salt	***Cleaning/Shelling*****:** Seeds were boiled to remove the hard coat that enveloped them. ***Drying*****:** Seeds were sun-dried or roasted on a fire.	-	***Pounding/Milling*****:** Seeds were ground with a manual grinding machine (*dastar*).	***Dough*****:** Flour was mixed with water and a small amount of wheat flour.
Iraq	Kurdistan/Shanidar, Barzan	Flatbread	*Astuk* (S)	*Quercus aegilops* Scop. (=*Quercus cerris* L.)	Acorn flour Water Honey or Ground mulberry Salt	-	***Boiling and drying*****:** Fruits were washed, boiled and dried to remove the coat and reduce the bitterness of the seeds.	***Pounding/Milling*****:** Dried seeds were ground using two big flat rocks (*juni*).	***Dough*****:** Flour was mixed with water, honey or ground white mulberry.
Iraq	Kurdistan/Hawraman	Flatbread	*Nani* *baru* (B)	*Quercus aegilops* Scop. (=*Quercus cerris* L.)	Acorn flour Water Salt	***Cleaning/Shelling*****:** Fruits were boiled to remove the hard coat and skin. ***Drying*****:** Seeds were sun-dried.	-	***Pounding/Milling*****:** Dried seeds were ground with a manual grinding machine (*dastar*).	***Dough*****:** Flour was mixed with water and baked.

^a^ Recorded folk name(s): A (Arabic), B (Badini dialect, Kurdish), C (Calabrian dialect, Italian), D (Dari), L (Lori), Sa (Sardinian dialect, Italian) and So (Sorani dialect, Kurdish).

**Table 3:** Comparison of acorn-based ingredients and preparation processes of acorn-based products documented in the Mediterranean basin, Central Asia and the Middle East.

		Mediterranean ^a^	Central Asia and Middle East ^b^
Product category	*Bread*	AL/CA/SY	-
	*Flatbread*	-	AF/IRN/IRQ
	*Other*	SA	-
Taxon	*Bitter varieties*	AL	AF
	*Sweet varieties*	SA/CA/SY	AF/IRN/IRQ
Ingredients	*Acorn flour*	SY	AF/IRN
	*Acorn flour and cereals/legumes*	AL/CA	IRQ
	*Acorn, clay and ash*	SA	-
Drying and cleaning/shelling	*Boiling and drying*	AL	IRQ
	*Roasting*	SA/CA/SY	AF/IRN/IRQ
	*Sun-drying*	SA/CA	IRN/IRQ
Leaching techniques	*Boiling*	SA	-
	*Boiling and drying*	AL/CA/SY	AF/IRQ
	*Roasting*	CA	-
	*Pounding and washing*	-	AF/IRN
Breadmaking (cooking)	*Boiling*	SA	-
	*Metal griddle/Pan*	-	AF/IRN
	*Vertical oven*	-	-
	*Wooden oven*	AL/CA/SY	IRQ

^a^ AL (Algeria), CA (Calabria), SA (Sardinia), SY (Syria). ^b^ AF (Afghanistan), IRN (Iran) and IRQ (Iraq).

## Data Availability

The data presented in this study are available on request from the corresponding author.
